# Prevalence, correlates, and network analysis of depression and its associated quality of life among ophthalmology nurses during the COVID-19 pandemic

**DOI:** 10.3389/fpsyg.2023.1218747

**Published:** 2023-08-24

**Authors:** Zi-Han Liu, Yue Li, Zi-Rong Tian, Yan-Jie Zhao, Teris Cheung, Zhaohui Su, Pan Chen, Chee H. Ng, Feng-Rong An, Yu-Tao Xiang

**Affiliations:** ^1^Department of Psychiatry, The Fifth Affiliated Hospital of Sun Yat-sen University, Zhuhai, Guangdong, China; ^2^Department of Nursing, Beijing Tongren Hospital, Capital Medical University, Beijing, China; ^3^The National Clinical Research Center for Mental Disorders & Beijing Key Laboratory of Mental Disorders, Beijing Anding Hospital & the Advanced Innovation Center for Human Brain Protection, Capital Medical University, Beijing, China; ^4^School of Nursing, Hong Kong Polytechnic University, Kowloon, Hong Kong SAR, China; ^5^School of Public Health, Southeast University, Nanjing, China; ^6^Unit of Psychiatry, Department of Public Health and Medicinal Administration, & Institute of Translational Medicine, Faculty of Health Sciences, University of Macau, Macao, Macao SAR, China; ^7^Centre for Cognitive and Brain Sciences, University of Macau, Macao, Macao SAR, China; ^8^Department of Psychiatry, The Melbourne Clinic and St Vincent’s Hospital, University of Melbourne, Richmond, VIC, Australia

**Keywords:** COVID-19, depression, ophthalmology, nurse, network analysis

## Abstract

**Background:**

Nurses in Ophthalmology Department (OD) had a high risk of infection during the novel coronavirus disease 2019 (COVID-19) pandemic. This study examined the prevalence, correlates, and network structure of depression, and explored its association with quality of life (QOL) in Chinese OD nurses.

**Methods:**

Based on a cross-sectional survey, demographic and clinical data were collected. Depression was measured with the 9-item Self-reported Patient Health Questionnaire (PHQ-9), and QOL was measured using the World Health Organization Quality of Life Questionnaire-brief version (WHOQOL-BREF). Univariate analyses, multivariate logistic regression analyses, and network analyses were performed.

**Results:**

Altogether, 2,155 OD nurses were included. The overall prevalence of depression among OD nurses was 32.71% (95%CI: 30.73–34.70%). Multiple logistic regression analysis revealed that having family or friends or colleagues who were infected (OR = 1.760, *p* = 0.003) was significantly associated with higher risk of depression. After controlling for covariates, nurses with depression reported lower QOL (*F*_(1, 2,155)_ = 596.784, *p* < 0.001) than those without depression. Network analyses revealed that ‘*Sad Mood’*, ‘*Energy Loss’* and *‘Worthlessness’* were the key central symptoms.

**Conclusion:**

Depression was common among OD nurses during the COVID-19 pandemic. Considering the negative impact of depression on QOL and daily life, regular screening for depression, timely counselling service, and psychiatric treatment should be provided for OD nurses, especially those who had infected family/friends or colleagues. Central symptoms identified in network analysis should be targeted in the treatment of depression.

## 1. Introduction

Since the novel coronavirus disease 2019 (COVID-19) emerged in December 2019, over 676 million people were infected globally and about 6.9 million people had died from COVID-19 as of March 10, 2023 (The Johns Hopkins Coronavirus Resource Center, 2023). The high rate of infection has caused immense workload and challenges on healthcare systems worldwide. Frontline health caregivers have played a critical and direct role in caring for infected patients and their families, which put them at a higher risk of infection compared to most of other populations (Chidiebere Okechukwu et al., 2020; Huang et al., 2020; Shah et al., 2021). Among the frontline workers, nurses, who are primarily responsible for infected patients, face a higher risk of infection than other healthcare professionals due to their frequent and prolonged physical contact with patients (Fawaz et al., 2020). In particular, nurses working in Emergency Departments (ED) and fever clinics face the highest risk of infection as they are managing COVID-19 patients who first present for care (Lu et al., 2015; An et al., 2020). However, preliminary evidence indicated that eyecare providers could also be exposed to substantial COVID-19 infection risk (Grossman et al., 2020; Seah et al., 2020). In fact, OD nurses faced high risk of infection in daily clinical practice due to transmission of COVID-19 through ocular fluids from slit-lamp examination and ophthalmoscopy (Williams et al., 2020), and respiratory droplets due to close physical contact (Sadhu et al., 2020). A study found that around 80% of ophthalmology health workers perceived they had a high risk of COVID-19 transmission because of the close proximity to patients and lack of Personal Protective Equipment (Xie et al., 2022) such as protective goggles (Minocha et al., 2020). Some studies found that conjunctivitis could be the first presentation of COVID-19 before the onset of usual symptoms like cough, fever, and fatigue (Seah and Agrawal, 2020; Daruich et al., 2020a, b); therefore, COVID-19 patients with only ocular symptoms might be presenting first to OD staff who were not aware of the transmission risk. A tragic example is Li Wenliang, an well-known OD health worker in Wuhan, who died from COVID-19 in January 2020 after coming into contact with a patient presenting with glaucoma as the primary complaint rather than typical COVID-19 symptoms (Parrish 2nd et al., 2020). Due to high work pressure caused by the COVID-19 pandemic, mental health problems, particularly depression, among frontline healthcare workers have become a major concern (Lee et al., 2022; Mercader Rubio et al., 2022). Several studies on depression among OD doctors during the pandemic have found a wide prevalence ranging from 32.6 to 65% (Almater et al., 2020; Khanna et al., 2020; Durmaz Engin et al., 2021; Grover et al., 2021). However, depression among OD nurses has not been adequately studied despite their higher infection risk due to prolonged close contact with patients (Chidiebere Okechukwu et al., 2020; Shah et al., 2021).

After the peak of the first COVID-19 wave in China in February of 2020, subsequent waves have occurred due to the rapid mutation of the virus and the waning of antibodies resulting from previous infections or vaccinations (Singh and Yi, 2021). This has resulted in significant impact on the work productivity, well-being, cognition, and mental health of healthcare workers. To develop effective public health interventions and strategies, it is important to examine COVID-19 related mental health problems among the vulnerable populations. Understanding the pattern of depression and its related factors among different healthcare workers is crucial for developing appropriate preventive strategies, treatment modalities, and management protocols.

Quality of Life (QOL) refers to the individual’s perception of their position in life in the context of their culture and value systems, and in relation to their goals, expectations, standards, and concerns, all of which could have an impact on their well-being. QOL is also associated with various factors such as economic status, personal relationships, and mental health (World Health Organization, 2023). Research has shown that the QOL of health professionals could affect the quality of medical services and even the prevention of infection outbreaks (Kandula and Wake, 2021). Therefore, addressing the QOL of healthcare professionals is highly important.

In the past decade, network analysis has been widely used to examine the complex associations among individual symptoms of a disorder (Beard et al., 2016), which was based on partial correlations between symptoms (Epskamp et al., 2018b). Network analysis identifies highly central symptoms (defined as nodes) and symptom-symptom interactions (defined as edges), and generates network models in which central nodes are located at the center, while nodes with fewer connections are at the periphery (Mullarkey et al., 2019b). Identifying central nodes and the relevant influential edges using network analysis may be helpful to develop effective treatment and improve health outcomes (Hofmann et al., 2016). Previous studies have examined depression networks among various populations, including adolescents, older adults, and the general population (Zhao et al., 2021; Eli et al., 2022; Xie et al., 2022). For instance, among Chinese adolescents, “fatigue,” “depressed mood,” and “self-blame” were identified as the most central symptoms (Xie et al., 2022). Among the Wuhan residents during the COVID-19 pandemic, “fatigue,” “sad mood,” “guilt,” and “motor disturbances” were the most central symptoms (Zhao et al., 2021). As most studies on depression among ophthalmologists were based on total scale scores, it was previously not possible to examine the syndrome of depression at the symptom level.

This study thus examined the epidemiological patterns and correlates of depression among OD nurses during COVID-19 pandemic in China, constructed the network structure and identified the central symptoms of depression, and explored the relationship between depression and QOL. Based on previous studies and reports, we hypothesized that depression among OD nurses would be common, and certain factors would be significantly associated with depression (Almater et al., 2020; Durmaz Engin et al., 2021; Grover et al., 2021). We also hypothesized that depression would be negatively associated with overall QOL.

## 2. Methods

### 2.1. Setting and study sample

This nationwide survey in China was jointly conducted by the Chinese Nursing Association Psychiatry Branch, and the Chinese Nursing Society Ophthalmology Branch from March 15 to March 20, 2020 (i.e., after the peak of the first COVID-19 wave), using a snowball sampling method. At the time the survey, 81,554 people in China were infected and 3,312 had died (Chinese Health Commission, 2020).

Due to the risk of COVID-19 infection, face-to-face assessment were not adopted for safety reasons. Similar to other studies (Zou et al., 2020; Yuan et al., 2021; Liu et al., 2022), the WeChat-based QuestionnaireStar program, a commonly used application for epidemiological surveys (Li et al., 2016; Liu et al., 2022), was used for data collection. A WeChat-based Quick Response code (QR code) linked to the study invitation and questionnaire was distributed by the Chinese Nursing Society Ophthalmology Branch to all OD nurses working in public hospitals in China. Those OD nurses who agreed then participated in this study on an anonymous and voluntary basis. To be eligible, participants met the following inclusion criteria: (1) adults aged 18 years or above; (2) OD nurses working in Chinese public hospitals during the COVID-19 pandemic; (3) able to understand Chinese and provide electronic written informed consent. The protocol of this study was approved by the ethics committee of Beijing Anding Hospital, China.

### 2.2. Instruments

Basic demographic and clinical data, such as age, gender, marital status, educational level, working experience, duty shift, living circumstances, rank (junior/senior), hospital setting (primary/tertiary), working units (inpatient/outpatient department), current smoking status, and work experience during the 2003 SARS outbreak, were collected. Three additional standard questions were asked: (1) whether they were directly engaged in clinical services for COVID-19 patients; (2) whether they had families, friends or colleagues infected with COVID-19; and (3) whether the local COVID-19 confirmed cases were more than 500 in the province they lived in.

Due to the risk of COVID-19 infection, interviewer-rated measures on depression in a face-to-face interview were not used. The severity of depression was measured with the validated self-report 9-item Patient Health Questionnaire (PHQ-9)-Chinese version, which was one of the most widely used measure on depression in different populations during the pandemic (Liu et al., 2021; Stocker et al., 2021; Shevlin et al., 2022). For example, a meta-analysis showed that over half of studies on depression during the pandemic used the PHQ-9 (Liu et al., 2021). The PHQ-9 was chosen instead of other standardized measures because of its brevity and wide application across different Chinese populations (Wang et al., 2014; Li et al., 2021; Kong et al., 2022). PHQ-9 is also a rapid screening tool for depression in large-scale population surveys (Levis et al., 2019; Sun et al., 2020).

Each item of PHQ-9 is scored from 0 to 3, with the total score of 5 being the cut-off value for “having depression” (Kroenke et al., 2010). The nine PHQ items measure nine cluster of depressive symptoms in accordance with the diagnosis criteria of major depressive disorder in the *Diagnostic and Statistical Manual of Mental Disorders (DSM-IV)*, including ‘*Anhedonia’*, ‘*Sad Mood’*, ‘*Sleep Disturbance’*, ‘*Energy Loss’*, ‘*Appetite Change’*, ‘*Worthlessness’*, ‘*Concentration Difficulty’*, ‘*Psychomotor Issues’*, and *‘Suicidal Ideation’*(American Psychiatric Association, 2013; Wang et al., 2014; Levis et al., 2019; Sun et al., 2020). Specifically, the PHQ-9 total score of 5–9, 10–14, 15–19, ≥20 indicate ‘mild depression’, ‘moderate depression’, ‘moderate-to-severe depression’, and ‘severe depression’, respectively (Kroenke et al., 2010). Psychometric properties of the Chinese version of PHQ-9 were found to be satisfactory (Cronbach’s alpha = 0.89)(Chen et al., 2015). Global QOL was measured by the sum of the first two items on the global QOL derived from the World Health Organization Quality of Life Questionnaire-brief version (WHOQOL-BREF)-Chinese version (Harper and Power, 1998; Fang and Hao, 1999). Higher score indicates higher QOL (Skevington and Tucker, 1999).

### 2.3. Data analysis

Data were analysed using the IBM Statistical Package for Social Science (SPSS) software version 23.0. P–P plots were performed to test the normal distribution of continuous variables. We divided participants into two groups according to PHQ-9 total score, depression group (PHQ-9 ≥ 5) and non-depression group (PHQ-9 < 5). To compare the demographic and clinical variables between the two groups, Chi-square test and two samples independent sample t-test were used where appropriate. Multiple logistic regression analysis with the “Enter” method was conducted to examine the independent demographic and clinical correlates of depression, with depression as the dependent variable, and variables with *p* values of <0.05 in the univariate analyses as independent variables. To compare QOL between the two groups, analysis of covariance (ANCOVA) was conducted after controlling for all the potential confounders. Significant level was set as *p* value less than 0.05 (two-tailed).

### 2.4. Network estimation

Following previous studies (Mullarkey et al., 2019; Peng et al., 2023; Wang et al., 2023), we performed network analysis in the depression group (*N* = 705) to explore the central symptoms and key edges of depression among Chinese OD nurses. Network analyses were conducted using *bootnet* (Epskamp et al., 2018a) and qgraph (Epskamp et al., 2012) packages in R program (version 4.1.2). In the network analysis, each individual depressive symptom was defined as ‘*node’* and relationships between symptoms were defined as ‘*edges’* (Beard et al., 2016; Wang et al., 2020). In this study, the PHQ-9 depressive symptoms were included for network analyses. For network visualization, the thickness of edges represented the strength of associations between two nodes. The colours of the edge indicated the direction of the correlations (i.e., green represented positive correlations, while red represented negative correlations).

Following previous studies (Beard et al., 2016; Wang et al., 2020), the ‘estimateNetwork’ function in *bootnet* package (Epskamp et al., 2018a) was used to estimate the network structure, with ‘*EBICglasso*’ as default method and 0.5 as default tuning parameter. The network models were estimated using a sparse *Graphical Gaussian Model (GGM)* combined with graphical least absolute shrinkage and selection operator (LASSO) method (Friedman et al., 2008); model selection was based on the *Extended Bayesian Information Criterion (EBIC)* (Chen and Chen, 2008 Epskamp, 2016). The *GGM* method was applied to construct network models. Considering the large number of spurious edges produced due to latent variables (Epskamp et al., 2018b; Montazeri et al., 2020), to reduce the number of spurious edges and improve the interpretability of networks, the network models were regularized using the *LASSO*, which is a well-established method for regularization (Epskamp et al., 2018a). This algorithm could reduce small associations to zero by removing them from the model as potentially “false positive” edges (Friedman et al., 2008; Heeren et al., 2018; Epskamp et al., 2018a).

To assess the importance of each node in the network, centrality index ‘*Strength’* was calculated (Freeman, 1978; Borgatti, 2005; Opsahl et al., 2010; Pan and Liu, 2021), using the ‘centralityPlot’ function in *qgraph* package (Epskamp et al., 2012). ‘*Strength’* is the sum of absolute edge weights of all direct connections between a specific node and all other nodes, reflecting the importance of an individual symptom (Dalege et al., 2017).

### 2.5. Estimation of network accuracy, stability, and comparisons of edge and node strengths

Similar to previous studies (Beard et al., 2016; Belvederi Murri et al., 2020; Wang et al., 2020), the accuracy and stability of network were tested using R package *bootnet* (Epskamp et al., 2018a). First, to estimate the accuracy of edge weights, non-parametric bootstrapping (1,000 replicates, 8 cores) was performed to compute 95% confidence intervals (Dobson et al., 2021) of edge values. Second, bootstrapped difference test was used to determine significant differences between edges weight and node strengths. Finally, to determine the stability of centrality indices, case-dropping subset bootstrap (1,000 replicates, 8 cores) was performed to compute correlation stability coefficient (CS). A series of correlation values were calculated between the original centrality indices based on original sample, and subset centrality based on different subset of sample (e.g., 95% of the sample, 80, 70%, …, 25%) (Hevey, 2018; Epskamp et al., 2018a). The CS represents the maximum proportion of cases that could be drooped from the original sample, while the correlation coefficients between centrality indices based on the original networks, and centrality indices based on case-subset network could still reach at least 0.7 (default) (Epskamp et al., 2018a). Generally, the CS should not be lower than 0.25, and is preferably above 0.5 as recommended previously (Epskamp et al., 2018a).

## 3. Results

### 3.1. Demographic information

In total, 2,155 OD nurses met the inclusion criteria and completed the survey. The mean age of this sample was 34.32 ± 8.21 years, and 99% were female. The mean duration of work experience was 12.94 ± 9.07 years, and 57.5% were junior nurses. In addition, 10.4% of participants had work experience during SARS in 2003. Overall, 74.5% had worked in the inpatient department, 5.7% had infected family/friends/colleagues, and 6.5% had experience caring for infected patients. Table 1 shows the basic social-demographic characteristics of the participants.

**Table 1:** Demographic characteristics of nurses in the department of ophthalmology.

Categorical Variables	Total (*N* = 2,155)		No depression (*N* = 1,450)		Depression (*N* = 705)		*X* ^2^	df	*P*
	*N*	%	*N*	%	*N*	%			
Male gender	22	1.0	16	1.1	6	0.9	0.299	1	0.584
Married	1,619	75.1	352	24.3	521	73.9	0.844	1	0.358
College education and above	2,112	98	1,416	97.7	696	98.7	2.768	1	**0.096**
Living with family	1851	85.9	1,255	86.6	596	84.5	1.586	1	0.208
Junior nurses	1,239	57.5	825	56.9	414	58.7	0.648	1	0.421
Work experience during SARS	224	10.4	156	10.8	68	9.6	0.631	1	0.427
Working in tertiary hospitals	1750	81.2	1,171	80.8	579	82.1	0.583	1	0.445
Working in inpatient department	1,605	74.5	1,079	74.4	526	74.6	0.010	1	0.922
Shift duty nurses	1,273	59.1	850	58.6	423	60.0	0.373	1	0.541
Local COVID-19 cases ≥500	375	17.4	245	16.9	130	18.4	0.786	1	0.375
Having infected family/friends/colleagues	122	5.7	65	4.5	57	8.1	11.526	1	**0.001**
Taking care of infected patients	141	6.5	83	5.7	58	8.2	4.859	1	**0.027**
Current smoker	14	0.6	9	0.6	5	0.7	0.058	1	0.810

Continuous variables	Mean	SD	Mean	SD	Mean	SD	*t*	df	*P*
Age (years)	34.32	8.21	34.46	8.44	34.04	7.70	1.140	1515.8	0.254
Working experience (years)	12.94	9.07	13.07	9.31	12.68	8.55	0.984	1505.2	0.325
Total QOL score	6.61	1.52	7.11	1.39	5.59	1.25	25.464	1529.3	**<0.001**

Bolded values, *p* < 0.1; M, mean; SD, standard deviation; COVID-19, Corona virus disease 2019; SARS, severe acute respiratory syndrome; QOL, quality of life.

### 3.2. Prevalence, correlates of depression and descriptive information of symptoms

In the whole sample, the mean (SD) of the PHQ-9 total score was 3.60 (SD: 4.29). The prevalence of depression was 32.71% (95%CI: 30.73–34.70%; PHQ-9 total score of ≥5). Specifically, the prevalence rates of ‘mild depression’, ‘moderate depression’, ‘moderate-to-severe depression’, and ‘severe depression’ were 23.02% (95%CI: 21.27–24.87%), 6.73% (95%CI: 5.73–7.89%), 2.55% (95%CI: 1.94–3.33%) and 0.42% (95%CI, 0.21–0.83%), respectively.

The results of P–P plot showed that continuous variables (e.g., age, work experience, and total QOL scores) followed a normal distribution. Univariate analyses revealed that depression was significantly associated with college education and above (*p* = 0.096), having family/friends/colleagues who were infected (*p* = 0.001), and caring for infected patients (*p* = 0.027). Multiple logistic regression analysis revealed that having infected family/friends/colleagues (OR = 1.760, *p* = 0.003) was significantly associated with higher risk of depression (Table 2). Table 3 shows the mean, SD, minimum, maximum and frequency (Epskamp) of all the depressive symptoms as measured by PHQ-9 in the depression group. After controlling for variables with significant group differences in univariate analyses, ANCOVA revealed that nurses with depression reported lower QOL than non-depression group (*F*_(1, 2,155)_ = 596.784, *p* < 0.001).

**Table 2:** Independent correlates of depression by multiple logistic regression analysis.

Variables	Multiple logistic regression analysis		
	OR	95% CI	*P*
College education and above	1.814	0.864–3.810	0.116
Having infected family/friends/colleagues	1.760	1.209–2.561	**0.003**
Taking care of infected patients	1.332	0.932–1.904	0.115

*Bolded values, p < 0.05; CI, confidential interval; OR, odds ratio; QOL, quality of life*.

**Table 3:** Descriptive characteristics of depressive symptoms in the depression group (*N* = 705).

Symptoms	PHQ-9 item number	Mean	SD	Frequency (Epskamp)
Anhedonia	1	1.12	0.719	84.4
Sad mood	2	0.98	0.623	83.3
Sleep disturbance	3	1.42	0.827	90.8
Energy loss	4	1.36	0.674	96.6
Appetite change	5	1.11	0.785	79.3
Worthlessness	6	0.91	0.760	69.8
Concentration difficulties	7	0.94	0.779	71.1
Psychomotor issues	8	0.59	0.675	49.2
Self-harm ideation	9	0.30	0.583	24.3

### 3.3. Network structures, accuracy, and stability

**Figure 1:**
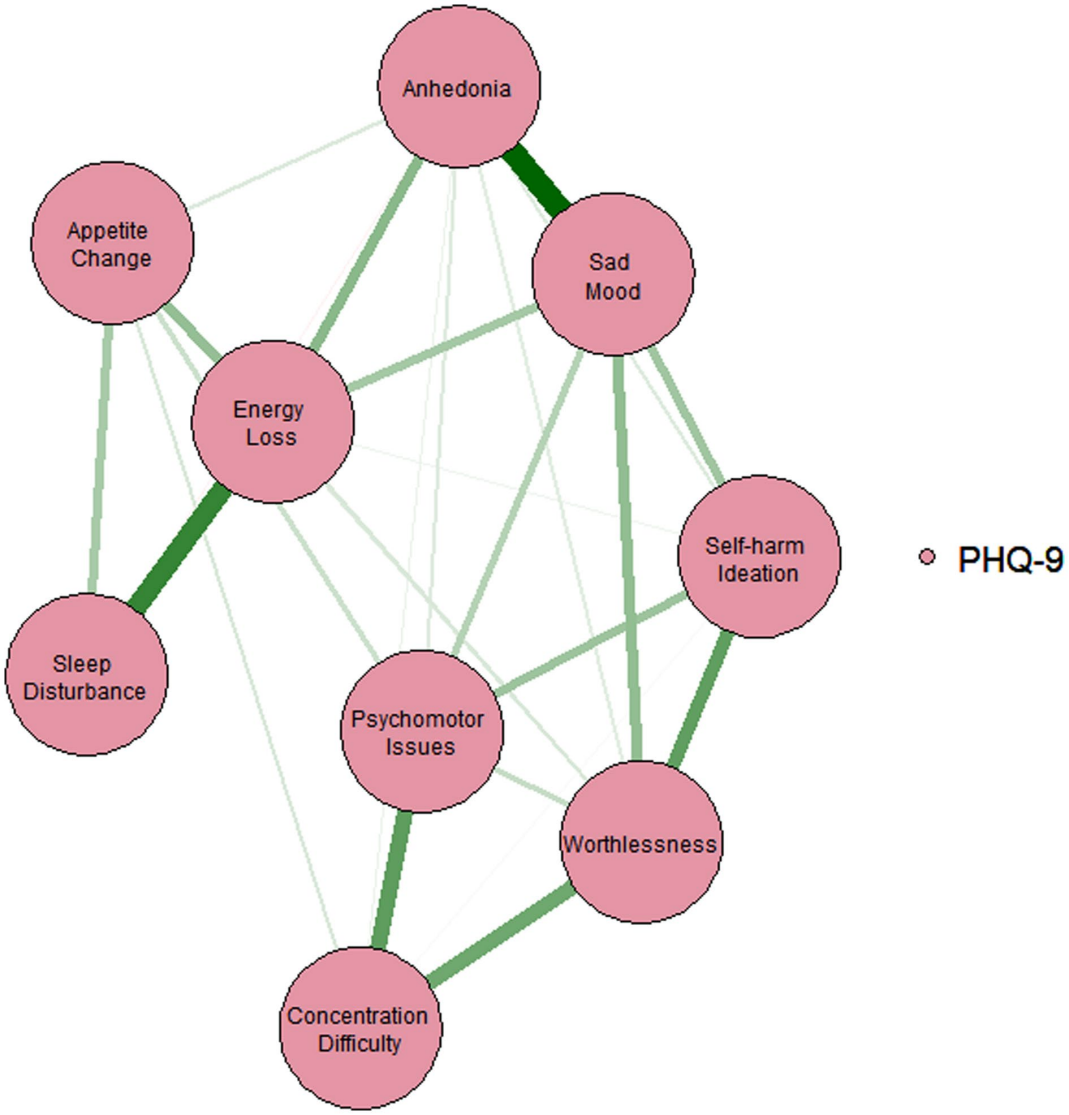
Estimated network model for depressive symptoms in the depression sample. The network models were estimated using the *‘EBICglasso’* model.

**Figure 2:**
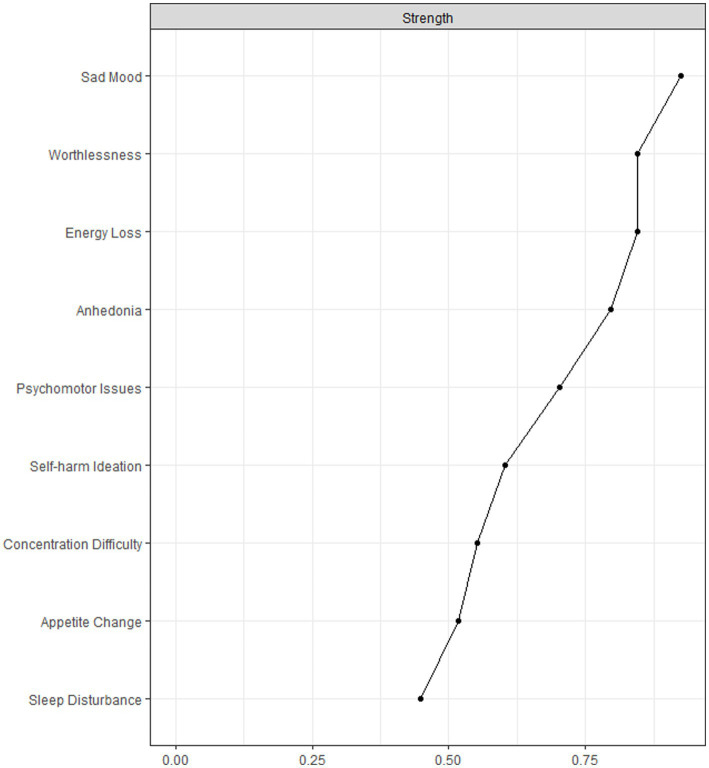
Centrality measures of all symptoms within the network in the depression sample. The figure shows centrality measure (i.e., strength) of all factors within the network (*z*-scores).

Following previous studies (Marchetti, 2019; Mullarkey et al., 2019), after checking for item informativeness (i.e., SD of the item) and item redundancy, we found that no item was poorly informative (i.e., 2.5 SD below the mean level of informativeness (Mullarkey et al., 2019), M_SD_ = 0.635 ± 0.141) or redundant with any other item (i.e., <25% of statistically different correlations). Therefore, all the PHQ-9 items were included in the analyses. The network of depressive symptoms in the depression group is shown in Figure 1. Figure 2 illustrates the centrality measure (i.e., *Strength*); the symptom *‘Sad Mood’* had the highest *strength*, followed by *‘Energy Loss’* and *‘Worthlessness’*.

**Figure 3:**
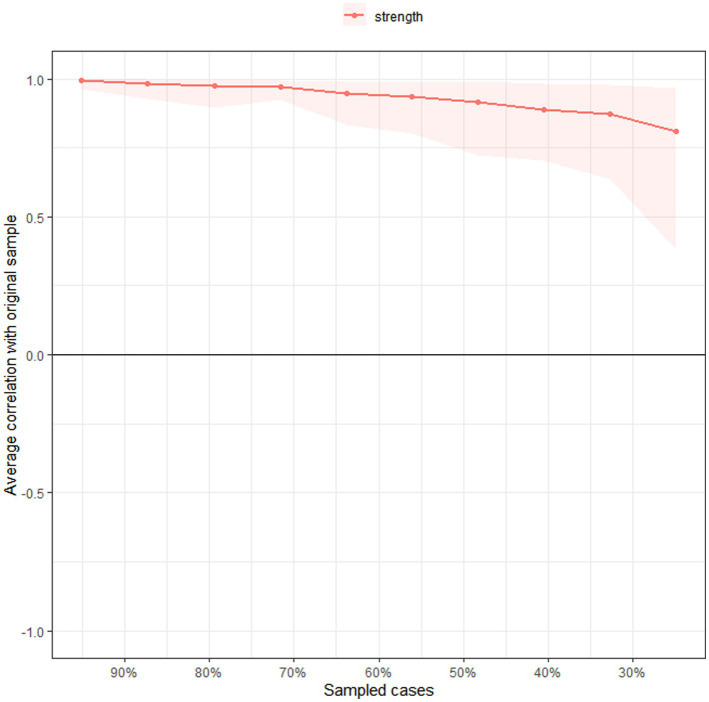
Stability of centrality indices by case dropping subset bootstrap. The *x*-axis represents the percentage of cases of the original sample used at each step. The *y*-axis represents the average of correlations between the centrality indices from the original network and the centrality indices from the networks that were re-estimated after excluding increasing percentages of cases.

Figure 3 shows the case-dropping subset bootstrap procedure, indicating that the value of strength remained stable even after dropping large proportions of the sample. The CS coefficients for ‘*strength’* was 0.675, indicating that 67.5% of the sample could be excluded with a higher correlation (*r* = 0.7) between the original whole sample and subset of sample, which suggested that the original results were robust and trustworthy. The *Bootstrap* 95%CI for edges within the network were narrow and the edge weights were consistent with the bootstrapped sample, suggesting that the precision of the edges was acceptable, with smaller CIs indicating more accurate estimation of the edges (Supplementary Figure S1). The bootstrapped difference test revealed that most of edge weights and node strengths were statistically significant from one another in the individual comparisons (Supplementary Figures S2, S3). For edge comparison, ‘Anhedonia-Sad Mood’, and ‘Sleep Disturbance-Energy Loss’ were the strongest edges that were statistically stronger than most of the other edges (Supplementary Figure S3).

## 4. Discussion

This was the first study that investigated the prevalence of depression, associated factors and impact on QOL among OD nurses in public hospitals in China during the peak of the first COVID-19 pandemic wave as well as the first network analysis of depressive symptoms in this population. In this study, we examined the prevalence, correlates, and network structure of depression among OD nurses and also performed a network analysis of depressive symptoms in this population. The findings provided valuable insights into the mental health and well-being of this overlooked group of healthcare professionals during the pandemic. This study focused specifically on OD nurses, who faced substantial risks of infection due to transmission through ocular fluids and respiratory droplets (Seah et al., 2020; Williams et al., 2020; Taha et al., 2022). The presence of COVID-19 patients with ocular symptoms who might not exhibit typical COVID-19 symptoms emphasizes the need for greater awareness and preparedness among ophthalmology healthcare professionals.

This study found that the overall depression prevalence among OD nurses was 32.71% (95% CI: 30.73–34.70%), which is comparable with a Chinese study (31.37%) using the same assessment tool among frontline health workers in China during COVID-19 pandemic (Zheng et al., 2020), and another study among ophthalmologists in India (32.6%) (Khanna et al., 2020). The high risk of infection from the close physical contact to conduct ophthalmoscopy in daily clinical practice could contribute to the high depression rate in our study (Williams et al., 2020; Durmaz Engin et al., 2021). Our findings suggest that even though OD was not a clinical unit with excessive workload burden like ED and fever clinics during the COVID-19 pandemic, OD nurses appeared to have an elevated risk of developing depression. Hence, mental health support should be implemented for OD nurses to address their risk of developing depression.

Although previous studies in frontline healthcare workers found that being directly involved in the care of COVID-19 patients was associated with a higher risk of depressive symptoms (Lai et al., 2020; Hruska et al., 2023), this study did not find any significant association between having depression and caring for infected patients after controlling for covariates. This could probably be due to use of different statistical methods (e.g., univariate vs. multivariate analyses) and different study stages of the COVID-19 pandemic (e.g., very early stage vs. middle stage). Instead, we found that having infected family/friends/colleagues was significantly associated with a higher risk of depression, which could be attributed to the uncertain prognosis of COVID-19 during the early/middle pandemic stages and the fear of being infected by family/friends/colleagues.

Our study also examined the QOL of OD nurses and its association with depression. The QOL of healthcare professionals is important due to its impact on the quality of medical services and infection prevention efforts (Kandula and Wake, 2021). Moreover, depression could affect sleep quality, social function and energy levels which could lead to a low quality of life (Malhi and Mann, 2018). As expected, we found that OD nurses with depression reported lower QOL than those without depression, which is similar to the previous findings (Malhi and Mann, 2018; Shao and Zhang, 2020; Sjöberg et al., 2020).

The network analysis of depressive symptoms provided valuable insights into the interconnected nature of these symptoms among OD nurses. Identifying the central symptoms and their connections could guide the development of targeted interventions. The network analysis revealed that “Sad Mood” was the most central symptom while “Worthlessness” and “Energy loss” were also other central symptoms. These findings are consistent with the notion that depression is characterized by low mood, lack of energy, and sadness, as well as one’s inability to enjoy life (Cui, 2015). Compared to other symptoms in the model, central symptoms are more important targets in treatments (Mullarkey et al., 2019). The network analysis also revealed that *‘Anhedonia-Sad Mood’*, and *‘Sleep Disturbance-Energy Loss’* were the strongest edges, which might indicate the potentially strong dynamic relationships between *‘Anhedonia’* and *‘Sad Mood’*, and between *‘Sleep Disturbance’* and *‘Energy Loss’*. Therefore, improving mood and energy levels could be possible by targeting the symptoms of anhedonia and sleep disturbances in clinical management. For example, optimizing the irregular working schedules might improve the sleep quality among nurses and reduce the symptom of ‘Energy Loss’. This study findings support the implementation of guidelines and strategies such as appropriate patient triage, sufficient PPE supply, and professional infection control training to protect OD nurses from the high infection risk during COVID-19, and decrease their anxiety and sense of vulnerability (Borrelli et al., 2020; Du et al., 2020; Romano et al., 2020; Yu et al., 2020). Another important implication of our findings is the need for accessible and effective mental health services for OD nurses. These interventions could include psychological counselling, stress management programs, and peer support groups that targets the unique difficulties and challenges faced by OD nurses during the pandemic. Furthermore, promoting a supportive work environment that encourages open discussions about mental health, reduces the stigma of mental disorders, and provides resources for help seeking (Jackson, 2021).

The strengths of this study included the relatively large sample size, use of standard instrument on depression and QOL, and the sophisticated network analysis that could analysis the inter-relationships between different depressive symptoms. However, several limitations should be acknowledged. First, the cross-sectional design of the study limits our ability to establish causal relationships between variables. Longitudinal studies are needed to understand the dynamic nature of depression and its impact on the QOL of OD nurses over time. Second, our study focused on OD nurses in China only, and thus may not be generalizable to nurses in other healthcare settings or different countries. Future research should include a more diverse sample to enhance the external validity of the findings. Third, most of the study sample were females, which reflected the actual gender distribution among healthcare workers in China (Han et al., 2021 National Health Commission of China, 2022). Fourth, for logistical and safety reasons, self-report measures were used to assess depression and QOL, which were subject to individual biases and might not fully capture the complexity of these constructs. The inclusion of interviewer-administered assessments and objective measures would strengthen the validity of the results in future studies. Additionally, as only global QOL was measured in this study, there was a lack of data on individual QOL domains. Hence, the network analysis of depressive symptoms and QOL domains was not performed. Fifth, as this was a nationwide online survey, hospital and provincial distribution of the study sample was not recorded. Finally, some variables related to depression were not examined, such as social support and pre-existing psychiatric illnesses.

In conclusion, this study found a high prevalence of depression among OD nurses during the first wave of the COVID-19 pandemic in China. The findings highlighted the need for targeted interventions, support programs, and a supportive work environment to address the mental health needs of this vulnerable group of healthcare professionals. Considering the negative impact of depression on QOL and daily life, regular screening for depression, timely counselling service, and psychiatric treatment should be provided for OD nurses, especially those who had infected family/friends or colleagues. Moreover, interventions for depression, such as cognitive behavioral therapy, should target the central symptoms (e.g., *‘Sad Mood’, ‘Worthlessness’*, and *‘Energy Loss’*) identified in the network analysis. These findings might have implications for the mental health support and well-being of healthcare professionals globally during the pandemic.

## Data availability statement

The datasets presented in this article are not readily available because the ethics committee of Beijing Anding Hospital that approved the study prohibits the authors from disseminating the research dataset of clinical studies publicly. Requests to access the datasets should be directed to xyutly@gmail.com.

## Ethics statement

The studies involving human participants were reviewed and approved by the ethics committee of Beijing Anding Hospital, China. The patients/participants provided their written informed consent to participate in this study.

## Author contributions

F-RA and Y-TX: conception and design. YL, Z-RT, and Y-TX: administrative support. YL, Z-RT, F-RA, and Y-TX: provision of study materials or patients. YL, Z-RT, Y-JZ, PC, and F-RA: collection and assembly of data. Z-HL, TC, ZS, and F-RA: data analysis and interpretation. Z-HL, CN, and Y-TX: manuscript writing and revision. Z-HL, YL, Z-RT, Y-JZ, TC, ZS, PC, CN, F-RA, and Y-TX: final approval of manuscript.

## Funding

The study was supported by the National Science and Technology Major Project for investigational new drug (2018ZX09201-014), the Beijing Hospitals Authority Clinical Medicine Development of special funding support (XMLX202128), and the University of Macau (MYRG2019-00066-FHS; MYRG2022-00187-FHS).

## Conflict of interest

The authors declare that the research was conducted in the absence of any commercial or financial relationships that could be construed as a potential conflict of interest.

## Publisher’s note

All claims expressed in this article are solely those of the authors and do not necessarily represent those of their affiliated organizations, or those of the publisher, the editors and the reviewers. Any product that may be evaluated in this article, or claim that may be made by its manufacturer, is not guaranteed or endorsed by the publisher.
