# Supplementary material for: Prevalence, correlates, and network analysis of depression and its associated quality of life among ophthalmology nurses during the COVID-19 pandemic
Source: Front Psychol. 2023 Aug 24;14:1218747. doi: 10.3389/fpsyg.2023.1218747 (PMC10484007; doi:10.3389/fpsyg.2023.1218747)
Supplement: Supplementary file 1 [file Table_1.DOCX]

**Supplementary figure 1: Bootstrapped confidence intervals of edge weights.** The black dots indicate the values of each edge weight, ordered from the highest to the lowest value. The gray area represents the 95% Confidence Intervals of edge weights, estimated with the non-parametric bootstrap procedure (*Bootnet* package). Wide intervals indicate lower stability and narrow intervals indicate higher stability.


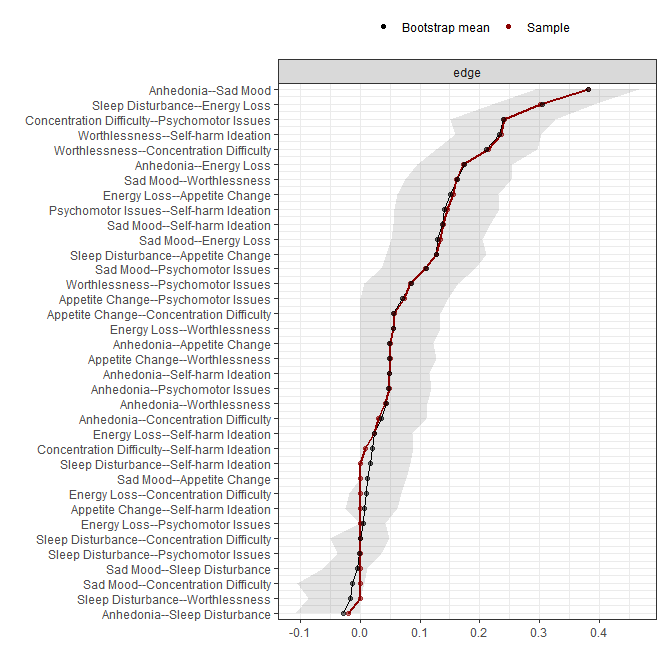


This result showed that the edge weights in the current sample were consistent with the bootstrapped sample, especially the connections with larger weights (i.e., Anhedonia-Sad Mood, Sleep Disturbance-Energy Loss, Concentration Problem-Motor), indicating that the current network structure was stable.

**Supplementary figure 2: Estimation of node strength difference by bootstrapped difference test.** Bootstrapped difference tests between node strength of factors. Gray boxes indicate nodes that do not significantly differ from one-another. Black boxes represent nodes that differ significantly from one another (α = 0.05). White boxes show the values of node strength.


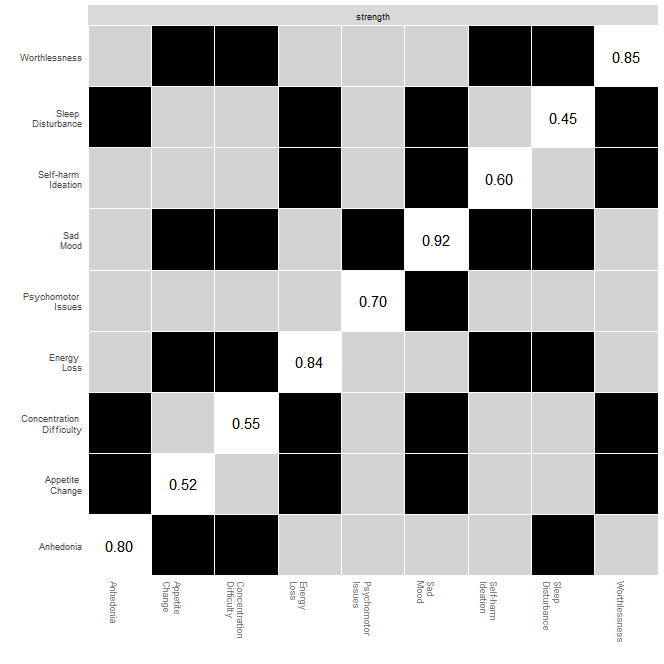


**Supplementary figure 3: Estimation of edge weight difference by bootstrapped difference test.** Bootstrapped difference tests between edge weights in the network. Gray boxes indicate edges that do not significantly differ from one-another. Black boxes represent edges with significant difference from one another (α = 0.05). Blue boxes in the edge-weight plot indicate positive correlations.

**
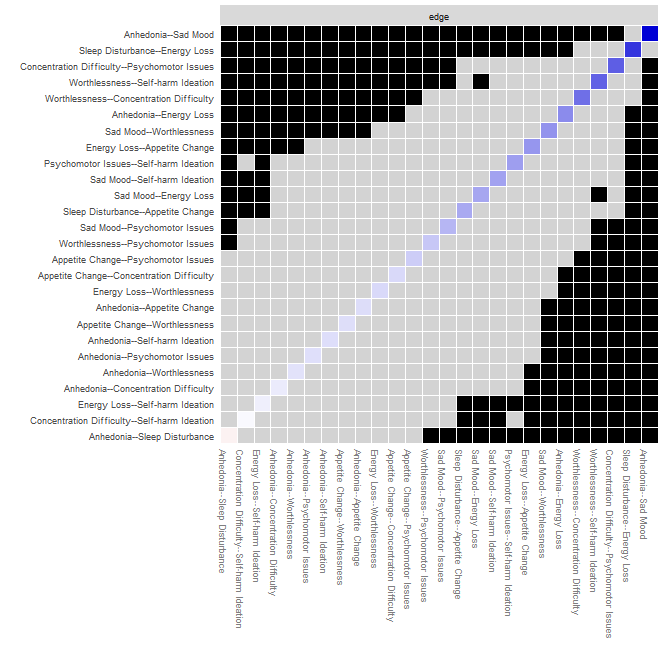
**
